# Regeneration of Pancreatic Non-β Endocrine Cells in Adult Mice following a Single Diabetes-Inducing Dose of Streptozotocin

**DOI:** 10.1371/journal.pone.0036675

**Published:** 2012-05-07

**Authors:** Yanqing Zhang, Yuan Zhang, Robert N. Bone, Wanxing Cui, Ji-Bin Peng, Gene P. Siegal, Hongjun Wang, Hongju Wu

**Affiliations:** 1 Department of Obstetrics and Gynecology, University of Alabama at Birmingham, Birmingham, Alabama, United States of America; 2 Department of Medicine, University of Alabama at Birmingham, Birmingham, Alabama, United States of America; 3 Department of Pathology, University of Alabama at Birmingham, Birmingham, Alabama, United States of America; 4 Department of Surgery, University of Alabama at Birmingham, Birmingham, Alabama, United States of America; 5 Department of Cell Biology, University of Alabama at Birmingham, Birmingham, Alabama, United States of America; 6 Department of Surgery, Medical University of South Carolina, Charleston, South Carolina, United States of America; 7 Department of Pathology, Guangzhou Medical University, Guangzhou, Guangdong Province, China; 8 Department of Medicine, Tulane University, New Orleans, Louisiana, United States of America; University of Bremen, Germany

## Abstract

The non-β endocrine cells in pancreatic islets play an essential counterpart and regulatory role to the insulin-producing β-cells in the regulation of blood-glucose homeostasis. While significant progress has been made towards the understanding of β-cell regeneration in adults, very little is known about the regeneration of the non-β endocrine cells such as glucagon-producing α-cells and somatostatin producing δ-cells. Previous studies have noted the increase of α-cell composition in diabetes patients and in animal models. It is thus our hypothesis that non-β-cells such as α-cells and δ-cells in adults can regenerate, and that the regeneration accelerates in diabetic conditions. To test this hypothesis, we examined islet cell composition in a streptozotocin (STZ)-induced diabetes mouse model in detail. Our data showed the number of α-cells in each islet increased following STZ-mediated β-cell destruction, peaked at Day 6, which was about 3 times that of normal islets. In addition, we found δ-cell numbers doubled by Day 6 following STZ treatment. These data suggest α- and δ-cell regeneration occurred rapidly following a single diabetes-inducing dose of STZ in mice. Using *in vivo* BrdU labeling techniques, we demonstrated α- and δ-cell regeneration involved cell proliferation. Co-staining of the islets with the proliferating cell marker Ki67 showed α- and δ-cells could replicate, suggesting self-duplication played a role in their regeneration. Furthermore, Pdx1^+^/Insulin^−^ cells were detected following STZ treatment, indicating the involvement of endocrine progenitor cells in the regeneration of these non-β cells. This is further confirmed by the detection of Pdx1^+^/glucagon^+^ cells and Pdx1^+^/somatostatin^+^ cells following STZ treatment. Taken together, our study demonstrated adult α- and δ-cells could regenerate, and both self-duplication and regeneration from endocrine precursor cells were involved in their regeneration.

## Introduction

The islets of Langerhans within the pancreas play a pivotal role in maintaining glucose homeostasis. Each islet typically contains five endocrine cell types, which include glucagon-producing α-cells, insulin-producing β-cells, somatostatin-producing δ-cells, pancreatic polypeptide-producing PP-cells, and ghrelin-producing ε-cells. The prevailing model of embryonic endocrine cell development is that all islet cells arise from common precursors, and sequential activation of hormone-specific genes are the key to their differentiation [1], [2], [3]. Although the reports on the precise appearance of the different lineages vary, it is generally found that most of the hormone-expressing cells that comprise the islets rapidly emerge around E13.5–E15.5 in mice [4], [5], [6], a period often referred to as the secondary transition in pancreatic development [1]. During neonatal life, endocrine pancreas undergoes substantial remodeling, which involves significant apoptosis, replication, and neogenesis of islet cells [7], [8], [9]. Islet mass grows into adulthood to match increased hormonal demand. In contrast, there is little change in islet mass in adults except in response to physiological/pathological changes such as pregnancy and obesity [10], [11], while adaptive islet cell proliferation is severely restricted in aged mice [12].

The etiology of Diabetes Mellitus is believed to be mainly caused by the lack of β-cells (Type 1 Diabetes) or deficiency in insulin signaling/secretion pathways (Type 2 Diabetes). Nonetheless, the non-β endocrine cells, especially the glucagon-producing α-cells, play a major counterpart and/or regulatory role to β-cells, thus are also crucial in the regulation of blood glucose. For instance, α-cells and β-cells have opposing effects in regulating blood glucose: glucagon activates glycogenolysis, ketogenesis and gluconeogenesis in the liver thus increasing blood glucose, while insulin stimulates the storage of glucose as glycogen in the liver and skeletal muscle and as triacylglycerol in adipose tissue thus reducing blood glucose. In addition, there is tight paracrine regulation between insulin and glucagon secretion: an increase in insulin suppresses glucagon secretion, and a decrease increases it, and vice versa [13], [14], [15]. The balance of the two opposing hormones is thus essential in maintaining blood glucose homeostasis. Additional paracrine regulation among the pancreatic endocrine hormones is also apparent. For example, somatostatin is a potent inhibitor of glucagon and insulin secretion [16], [17], [18]. Therefore, the topography of islets is essential in the coordinated responses of β- and non β-cells to minor changes in blood glucose, and its disruption results in the perturbation of glycemic control.

Regeneration of adult β-cells has attracted tremendous interest due to its potential therapeutic value for Type 1 Diabetes. Recently, scientists have found direct supporting evidence for adult β-cell regeneration, and begun to elucidate its mechanisms. Interestingly, the terminally differentiated β-cells in adults have been shown to be able to replicate/self-duplicate [19], [20], [21]. In fact, self-duplication appears to be the major mechanism for β-cell replenishment under normal physiological conditions. In addition, it has been demonstrated that β-cells can regenerate from stem cells located in pancreatic ducts or from progenitor cells residing inside murine islets [22], [23]. Moreover, following extreme β-cell loss, new β-cells can be transdifferentiated from glucagon-producing α-cells [24], [25]. In summary, β-cell regeneration in adults can occur by self-duplication, regeneration from progenitor cells or trans-differentiation from other cells in mammalian systems.

Regeneration of non-β endocrine cells in adults largely remains undefined. Previous studies have noted that the composition of α-cells increases in the pancreatic islets of diabetic patients and animal models [24], [26], [27], [28], [29], [30], implicating the regeneration of α-cells in adult animals and human patients as offsetting β-cell loss. For instance, glucagon^+^ cells were observed in pancreatic interlobular ducts of diabetic NOD mice, suggesting neogenesis of α-cells in these mice [27]. Alpha cell expansion has also been noted in adult mice whose β-cells were destroyed by specific toxins [24], [26], and in some type 2 diabetic patients and animal models [28], [29], [30]. The mechanisms or cellular origins of α-cell regeneration, however, remain to be investigated. Regeneration of other non-β cells such as δ-cells in adults is similarly not defined.

Our current study was aimed at exploring the regeneration of non-β endocrine cells in adult rodents. Based on previous observations, we hypothesized that adult non-β-cells could regenerate, and the regeneration could accelerate in the event of β-cell destruction. To test this hypothesis, we examined the β- and non-β cell composition in the islets of a commonly used diabetes animal model established with the β-cell specific toxin streptozotocin (STZ). Our data supported regeneration of α- and δ-cells in adults. Therefore, we further explored the potential mechanisms of their regeneration.

## Materials and Methods

### Ethics Statement

All animal procedures followed the guidelines set by the Institutional Animal Care and Use Committee at both the University of Alabama at Birmingham and Tulane University. The animal protocol approval numbers are 100308682 and 4254, respectively.

### Antibodies

Guinea pig anti-human Insulin polyclonal antibody (1∶100), mouse anti-BrdU monoclonal antibody (1∶500), rabbit anti-Glucagon (1∶200), rabbit anti-Somatostatin (1∶200), and rabbit anti-PPP (1∶300) polyclonal antibodies were purchased from Millipore Corp. (Billerica, Massachusetts). Mouse anti-glucagon monoclonal antibody (1∶500) was obtained from Sigma-Aldrich Corp. (St. Louis, MO), and a second rabbit anti-glucagon polyclonal antibody (1∶100) was obtained from Cell Signaling Technology (Danvers, Massachusetts). Rabbit anti-Ki67 (1∶500) and rabbit anti-Pdx1 (1∶500) polyclonal antibodies were purchased from Abcam Inc (Cambridge, Massachusetts). Rat anti-BrdU monoclonal antibody was obtained from AbD Serotec (Oxford, UK), and rat anti-somatostatin monoclonal antibody from Life Span Biosciences (Seattle, Washington). The Texas-Red (TR), FITC and CMCA-conjugated secondary antibodies, which include anti-mouse, anti-guinea pig, anti-rabbit, and anti-rat antibodies, were purchased from Jackson ImmunoResearch Laboratories Inc (West Grove, Pennsylvania). The nuclear dye Hoechst 33342 was obtained from Sigma-Aldrich Corp. (St. Louis, Missouri), and used at a final concentration of 2 µg/ml.

### Animals and treatment

C57BL/6 mice from either Jackson Labs (Chicago, Illinois) or Frederick Cancer Research (Hartford, Connecticut) were used in this study. To establish the STZ-induced diabetes state, 8–10 weeks old mice were fasted overnight, and treated with freshly prepared STZ solution at a dose of 130 mg/kg bodyweight via intraperitoneal (i.p.) injection. Four hours after injection, the mice received 200 µl of 20% glucose via intra-peritoneal injection to prevent hypoglycemia caused by sudden release of large amounts of insulin into the blood stream due to STZ-induced β-cell destruction. Normal mice that were not injected with STZ were used as the control. The blood glucose levels of each mouse were measured daily following 4–6 hours of fasting by the AlphaTRAK Blood Glucose Monitoring System (Abbott Laboratories, Abbott Park, Illinois). For BrdU incorporation, immediately following STZ injection, 1 mg/ml BrdU was added into the mice's drinking water and maintained until the mice were sacrificed. The mice were sacrificed at different time points post STZ injection, their pancreases harvested and processed for histological analysis. 8–10 mice were examined for each time point.

### Immunohistochemistry

The mouse pancreases were fixed in 10% buffered formalin solution overnight at room temperature, and then processed for paraffin embedding and sectioning. For immunohistochemistry assays, the pancreas sections were first deparaffinized with sequential incubation in xylene (4×2 minutes), 100% ethanol (2×1 minute), 95% ethanol (30 seconds), 70% ethanol (45 seconds) and deionized water (1 minute). Antigen retrieval was required for staining involving anti-BrdU, anti-Pdx1, and anti-Ki67 antibodies. This was conducted by boiling the pancreas slices in citrate buffer (pH 6.0) for 10–30 minutes, followed by natural cooling down to below 40°C, and washing with deionized water. Prior to antibody incubation, the tissue slices were permeablized with 0.25% Triton X-100 in Phosphate-Buffered Saline (PBS) for 30 minutes, and blocked in blocking solution (2% Glycine, 2% Bovine Serum Albumin, 5% Fetal Bovine Serum (FBS), 50 mM NH_4_Cl in PBS), for 1 hour, at room temperature. The slices were then incubated with the primary antibody diluted in 3% FBS in PBS, overnight at 4°C (for antigen retrieved samples) or for 2 hours at room temperature, followed by incubation with the corresponding secondary antibody that were conjugated to various fluorescent agents. For nuclear staining, Hoechst 33342 (2 µg/ml in PBS) was added to the slides after secondary antibody incubation, and incubated, for 10 minutes, at room temperature. The slices were then washed in PBS and water, air-dried, mounted with glass coverslip, and processed for fluorescence microscopy. Images were taken with CoolSNAP-HQ digital camera (Roper Scientific, Inc., Tucson, Arizona) that was attached to a Nikon Eclipse TE2000-U microscope (Nikon Instruments Inc., Melville, New York). Metamorph V7.7 software was used to collect images, and Adobe photoshop to edit and analyze the microscopic data.

### Quantitative determination of insulin and glucagon in mouse serum

Blood was collected at the time of pancreas harvest following 4–6 hours of fasting, allowed to clot at room temperature for 30 min, and then centrifuged at 3000 rpm for 15 minutes. Serum was transferred into new tubes, and stored at −20°C until further analysis. The insulin and glucagon concentrations in the mouse serum were determined using ultrasensitive ELISA kits for mouse insulin (cat# 80-INSMSU-E01) and glucagon (cat# 48-GLUHU-E01) from ALPCO Diagnostics (Salem, New Hampshire), respectively. The manufacturer's protocols were followed for both assays.

### Quantification of islet cells and statistical analysis

Following immunofluorescence staining of the islets and nuclei, the numbers of different cell types (α-, β-, δ-, BrdU^+^, Ki67^+^) cells and total islet cells in each islet were manually counted from the islet microscopy images stored in Adobe Photoshop. Nuclear staining was used to count the total cells in each islet. For each cell type, 20–35 representative islets from 4–5 mice were counted. The islets containing <30 cells were excluded. All statistical analyses were performed using GraphPad Prism biostatistics software (GraphPad Software, Inc, La Jolla, California). The data were expressed as Mean ± SEM (standard error of the mean). *P*<0.05 was considered statistically significant.

## Results

### STZ-mediated β-cell destruction

Based on previous observations, we hypothesized that, similar to the insulin-producing β-cells, non-β cells such as glucagon-producing α-cells and somatostatin-producing δ-cells in adults could regenerate, and the regeneration could accelerate in the event of β-cell loss. This study was thus designed to test this hypothesis using a widely employed diabetes model whereby a single-dose of STZ was used to achieve rapid β-cell destruction. For the study, we first performed a dose response curve in C57BL/6 mice (8–10 weeks old) via intraperitoneal (i. p.) injection with doses ranging from 100 mg to 180 mg of STZ per kilogram body weight (data not shown). We chose 130 mg of STZ per kilogram body weight as the optimal dose for this study because it resulted in hyperglycemia (blood glucose >300 mg/dL) in the mice within 2 days post injection, and allowed the mice to survive for approximately 2 weeks without any treatment.

**Figure 1:**
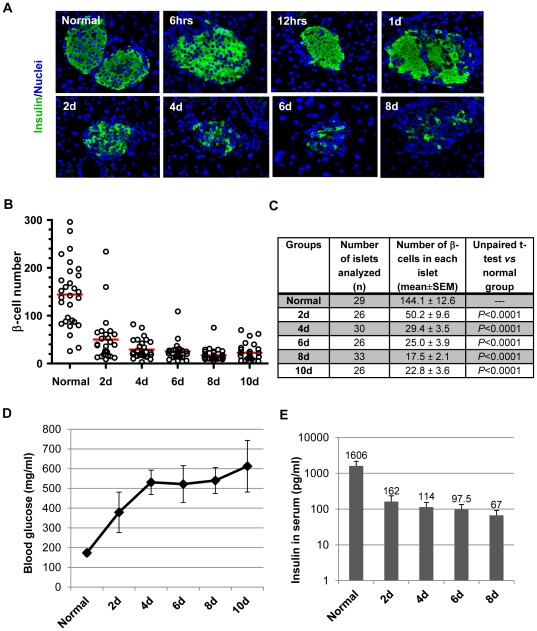
STZ-mediated β-cell destruction. Adult C57BL/6 mice were treated with STZ at a dose of 130 mg/kg body weight via i.p. injection, monitored for the development of hyperglycemia, and sacrificed at multiple time points post-STZ. Shown here are representative data. *A:* Anti-Insulin staining of the mouse pancreases showing β-cell loss following STZ treatment. *B:* Quantitative distribution of β-cells in each islet section. The number of β-cells in each islet was counted and plotted. Each circle represents one islet. For each group (normal, and 2, 4, 6, 8, and 10 days post-STZ), 26–33 representative islets from 4–5 mice were counted. The red line marks the mean value for each group. *C:* the statistical summary of β-cell distribution in islets from normal mice and STZ-treated mice. *D:* Blood glucose levels of the mice at different day post-STZ. Note: the blood glucose levels during the first 2 days were not included because STZ-induced hypoglycemia at early hours requiring a glucose injection to overcome hypoglycemia (see methods). *E:* Serum insulin concentrations in normal mice and those treated with STZ.

At select days after STZ treatment, the mouse pancreases were harvested and processed for immunohistochemical staining. Anti-insulin fluorescence staining of the pancreas slices showed acute β-cell loss within the first 24 hours post STZ injection (Fig. 1A). As shown in the representative microscopic images at 6 hrs, 12 hrs, and 24 hrs post injection, β-cells underwent necrosis, and lost their clear nuclei and cytoplasmic compartments. The remaining β-cells in the islets were evident but continued to decrease over the next few days. Quantification of the β-cells in each islet section showed β-cell number significantly decreased beyond Day 2 post-STZ treatment (2 d, 4 d, 6 d, 8 d, and 10 d) (Fig. 1B and 1C). The β-cell composition, which was calculated as the percentage of β-cells over the total number of cells in each islet, was reduced from about 70% in normal islets to less than 20% after STZ treatment (Table 1). In addition, the number of identifiable islets in each pancreas slice also decreased following STZ treatment. In normal mice, we could detect 6.5 islets/mm^2^ pancreatic slice. The density decreased to 4.3 islets/mm^2^ at Day 2, 3.4 islets/mm^2^ at Day 6, and 2.3 islets/mm^2^ at Day 8 post-STZ treatment. All of these data were consistent with the development of hyperglycemia in the STZ-treated mice (Fig. 1D). Also accordingly, the insulin levels in the serum of STZ-treated mice were significantly reduced, being about 10% of the normal level at Day 2 (2 d), and declining to less than 5% by Day 8 (8 d) post injection (Fig. 1E). Taken together, these characterizations confirmed STZ-mediated β-cell destruction and establishment of diabetes in the mice.

At select days after STZ treatment, the mouse pancreases were harvested and processed for immunohistochemical staining. Anti-insulin fluorescence staining of the pancreas slices showed acute β-cell loss within the first 24 hours post STZ injection (Fig. 1A). As shown in the representative microscopic images at 6 hrs, 12 hrs, and 24 hrs post injection, β-cells underwent necrosis, and lost their clear nuclei and cytoplasmic compartments. The remaining β-cells in the islets were evident but continued to decrease over the next few days. Quantification of the β-cells in each islet section showed β-cell number significantly decreased beyond Day 2 post-STZ treatment (2 d, 4 d, 6 d, 8 d, and 10 d) (Fig. 1B and 1C). The β-cell composition, which was calculated as the percentage of β-cells over the total number of cells in each islet, was reduced from about 70% in normal islets to less than 20% after STZ treatment (Table 1). In addition, the number of identifiable islets in each pancreas slice also decreased following STZ treatment. In normal mice, we could detect 6.5 islets/mm^2^ pancreatic slice. The density decreased to 4.3 islets/mm^2^ at Day 2, 3.4 islets/mm^2^ at Day 6, and 2.3 islets/mm^2^ at Day 8 post-STZ treatment. All of these data were consistent with the development of hyperglycemia in the STZ-treated mice (Fig. 1D). Also accordingly, the insulin levels in the serum of STZ-treated mice were significantly reduced, being about 10% of the normal level at Day 2 (2 d), and declining to less than 5% by Day 8 (8 d) post injection (Fig. 1E). Taken together, these characterizations confirmed STZ-mediated β-cell destruction and establishment of diabetes in the mice.

**Table 1:** Composition of α-, β- and δ-cells in islets from normal and STZ-treated mice.

Groups	% of α-cells in each islet	Unpaired t-test vs normal	% of β-cells in each islet	Unpaired t-test vs normal	% of δ-cells in each islet	Unpaired t-test vs normal
Normal	15.6±0.8	—	68.3±2.0	—	7.6±0.9	—
2 d	25.3±1.8	*p*<0.0001	37.6±2.0	*p*<0.0001	11.3±1.3	*p* = 0.027
4 d	36.7±2.3	*p*<0.0001	24.7±1.9	*p*<0.0001	16.5±1.6	*p*<0.0001
6 d	50.5±1.5	*p*<0.0001	17.9±1.3	*p*<0.0001	18.2±1.6	*p*<0.0001
8 d	44.8±1.9	*p*<0.0001	15.2±1.1	*p*<0.0001	19.5±1.8	*p*<0.0001
10 d	43.0±1.4	*p*<0.0001	18.7±1.3	*p*<0.0001	16.1±0.9	*p*<0.0001

The percentage of α-, β- and δ-cells was calculated against total cells (marked by nuclei staining) in each islet. 20–35 representative islets were counted for each group. The results were expressed as mean ± SEM. Unpaired t-tests were performed to compare the significance of the differences from the normal control. p<0.05 is defined as significantly different. (*: *p*<0.05, **: *p*<0.005, ***: *p*<0.0005).

### Rapid α-cell regeneration following STZ treatment

**Figure 2:**
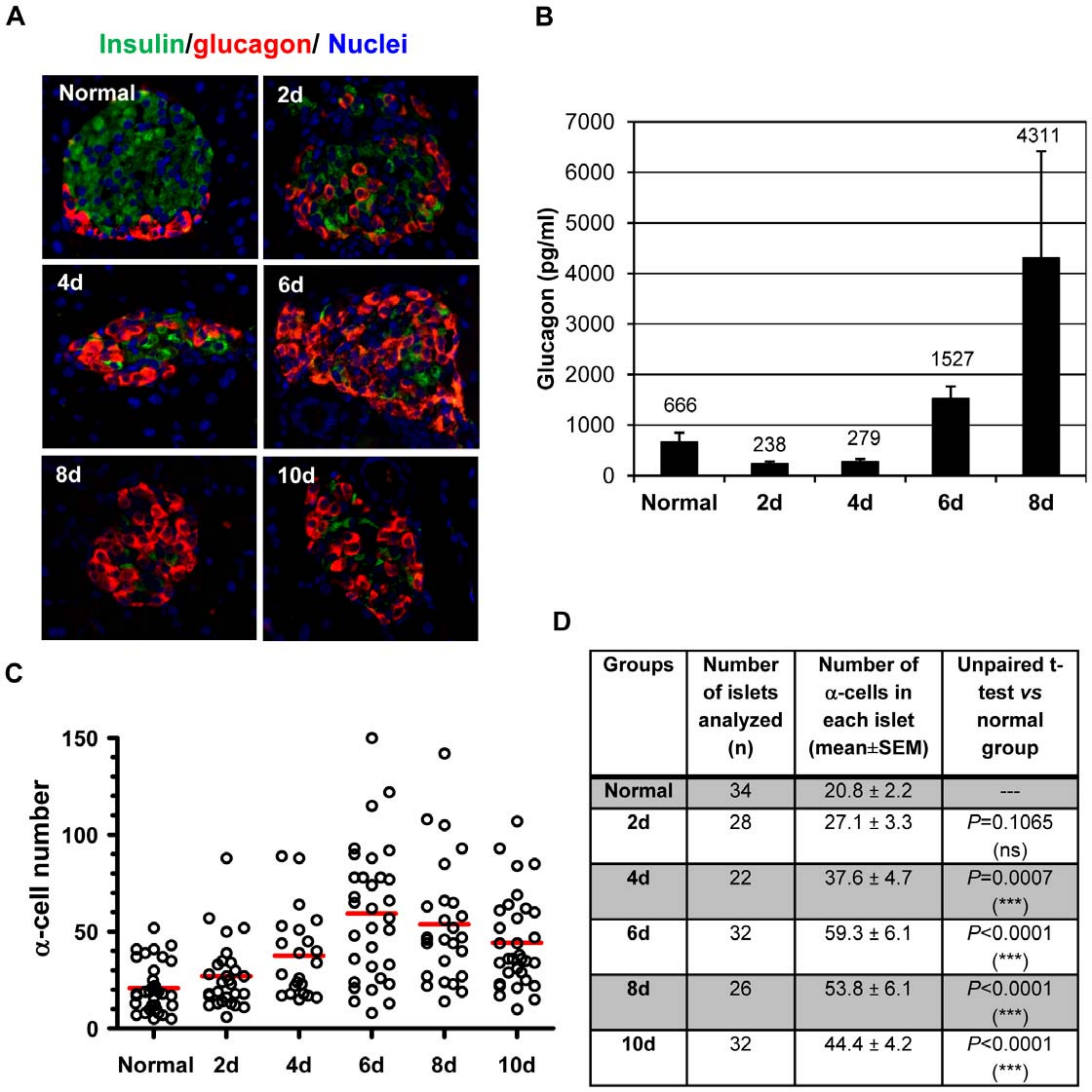
α-cell expansion following STZ-mediated β-cell destruction. *A:* Immunofluorescence staining showing α-cell expansion following STZ treatment. The pancreatic islets from normal and STZ-treated mice were co-stained with anti-Insulin (green) and anti-glucagon (red) antibodies. Hoechst 33342 was used to label nuclei (blue). *B:* Glucagon levels in the serum of normal and STZ-treated mice. *C:* Quantitative distribution of α-cells in each islet section. The number of α-cells in each islet was counted and plotted in the same way as described in Fig. 1B. 22–34 representative islets from 4–5 mice were counted. The red line marks the mean value for each group. *D:* the statistical summary of α-cell distribution in islets from normal mice and STZ-treated mice. *** indicates *p*<0.001; ns: not significant.

Next, we examined the distribution of α-cells in the pancreases of STZ-treated mice. Triple immunofluorescence staining with anti-insulin, anti-glucagon and a nuclear dye Hoechst 33342 showed that, in normal mouse islets, α-cells were localized at the periphery/mantle zone and β-cells in the core of the islets, which is consistent with previous observations [31], [32]. Two days following STZ injection, α-cells started to expand into the core area, and dominated the islets by Day 6 (Fig. 2A). Quantification of the α-cells in each islet section showed a rapid increase in α-cell numbers following STZ treatment, which became significantly different from normal islets by Day 4, and peaked at Day 6 (Fig. 2C and 2D). The mean α-cell number in each islet section was nearly doubled at Day 4, and tripled at Day 6 compared to that in the normal islets (Fig. 2D). The mean α-cell numbers per islet at Day 8 and Day 10 slightly decreased from the peak value, but were still more than 2 times of that seen in normal islets. This may be explained by the general toxicity related to the high blood glucose levels at these points. The rapid α-cell expansion was confirmed with 3 different glucagon antibodies that were from different commercial sources (see methods). Furthermore, the α-cell composition in each islet also changed accordingly (Table 1). The normal mouse islets contain about 15% of α-cells. Six days following STZ treatment, the α-cells constituted more than 50% of each islet (Table 1). Taken together, the rapid increase of α-cells in each remaining islet following STZ treatment argued for the regeneration of α-cells.

**Figure 3:**
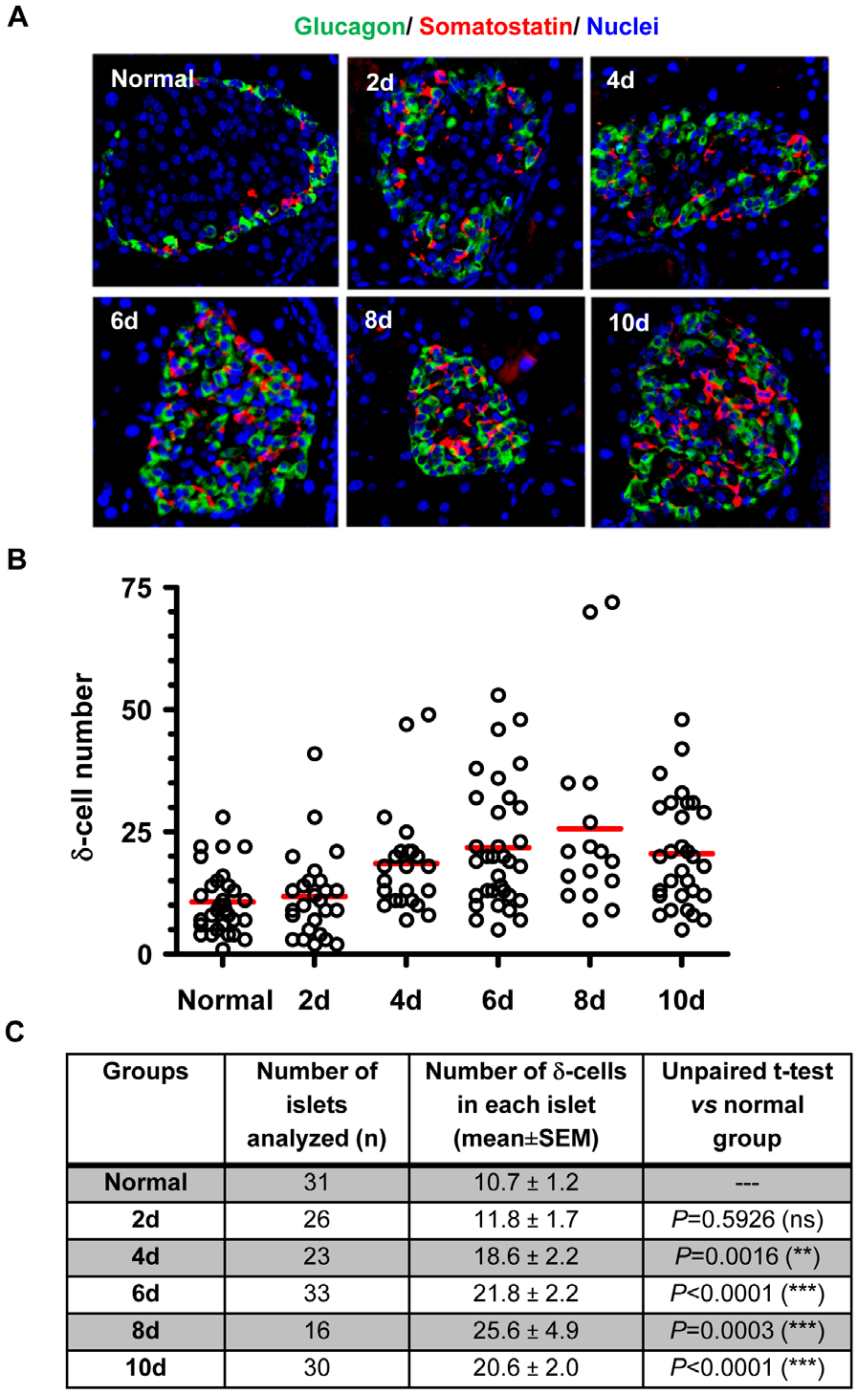
The distribution of α-cells and δ-cells following STZ treatment. The pancreatic islets from normal and STZ-treated mice were co-stained with anti-glucagon (green) and anti-somatostatin (red) antibodies. *A:* representative images showing δ-cells expanded in a similar pattern to α-cells following STZ treatment. *B:* Quantitative distribution of δ-cells in each islet group. The quantification of δ-cells was obtained the same way as described for α-cells and β-cells in Figures 1 and 2. The redlines represent the mean values for each islet group. C: the statistical summary of δ-cell distribution in islets from normal mice and STZ-treated mice. The mark ** indicates p<0.005, and *** indicates p<0.0005; ns: not significant.

Interestingly, although α-cell expansion started 2 days after STZ treatment, the glucagon concentration in serum decreased at Day 2 before gradually increasing, and became much higher than normal level by Day 6 (Fig. 2B). In a representative experiment, serum glucagon level reached to about 2 times of normal at Day 6, and more than 6 times at Day 8 (Fig. 2B). This is not surprising because glucagon secretion is highly regulated—it is normally inhibited by insulin, somatostatin and high blood glucose. During the first few days following STZ treatment, serum glucagon levels were low probably because of the release of a large amount of insulin from the dead β-cells, and the higher-than normal blood glucose. Later on, with the decrease of serum insulin concentration and increase of α-cells, insulin could no longer efficiently suppress glucagon secretion, thus serum glucagon levels were high even in the presence of hyperglycemia. In fact, the elevated glucagon levels could have also contributed to the development of hyperglycemia as it is known that glucagon stimulates glucose production from the liver.

### Expansion of δ-cells and PP cells following STZ treatment

We further investigated whether other non-β cells in the pancreatic islets of adult mice regenerated following STZ-mediated β-cell destruction. We first examined the distribution of somatostatin-producing δ-cells in the remaining islets of STZ-treated mice. Mouse islets normally contain 5–10% δ-cells which are localized to the islet periphery (Table 1). After a single diabetes-inducing dose of STZ, the distribution of δ-cells changed within days, and gradually became more centrally localized (Fig. 3A). Quantitative analysis showed δ-cell composition in the remaining islets also increased. The average number of δ-cells in each islet doubled by Day 6 compared to that in normal mouse islets (Fig. 3B and 3C), and constituted about 20% of the total cells in each islet (Table 1). These data thus also support the regeneration of δ-cells in the adult mice following STZ treatment. Co-staining of -cells and δ-cells suggested the two cell types followed a very similar pattern of expansion, although there were more α-cells than δ-cells in the remaining islets (Fig. 3A).

**Figure 4:**
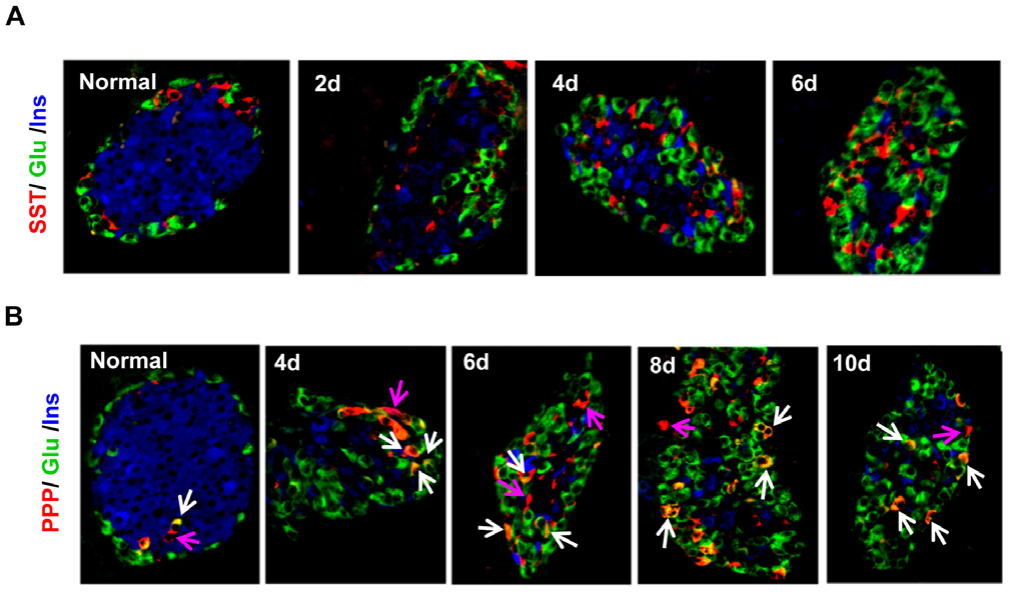
Islet architectural changes following STZ-mediated β-cell loss and non-β cell regeneration. *A:* Distribution of α-, β- and δ-cells as assessed by triple staining with anti-insulin (blue), anti-glucagon (green) and anti-somatostatin (red) antibodies. The three cell types gradually lost their normal mantle-to-core zone organization, and became intermingled throughout the islets. *B:* Distribution of α-, β- and PP cells as assessed by anti-insulin (blue), anti-glucagon (green)) and anti-PPP (red) antibodies. Note: most regenerated PPP^+^ cells appeared to co-express glucagon (white arrows) following STZ treatment. This did not seem to be attributable to antibody cross-reaction because there were many individually stained α-cells and PP-cells (pink arrows).

We next examined the distribution of PP cells. PP cells express pancreatic polypeptide (PPP), and constitute less than 5% of all islet cells in normal mice. Some PPP^+^ cells also express glucagon (Fig. 4B). Following STZ treatment, an increasing presence of PPP^+^ cells was observed in the islets over time, arguing for the regeneration of PP cells. Interestingly, most PPP^+^ cells were also glucagon positive (Fig. 4B). This phenomenon did not appear to be due to antibody cross-reaction because there were many individually stained PP-cells and α-cells. The nature of the PPP^+^/Glucagon^+^ bi-hormonal cells remains to be further investigated. Nonetheless, it is safe to say they represent a different population of islet cells from the unihormonal α-cells or PP-cells. Presence of these bi-hormonal cells complicated the quantification of PP-cells and thus was not performed.

The spatial distribution of α-cells, δ-cells and PP cells changed dramatically following STZ-induced β-cell loss. To gain a more clear insight into the islet cytoarchitecture, we performed triple immunofluorescence staining using select islet cell markers. The staining of α-, β-, and δ-cells showed a clear change in islet cytoarchitecture, with a loss in the core-to-mantle organization that was seen in normal mouse islets. The non-β cells extended from the periphery into the core, and became intermingled with the remaining β-cells throughout the islets (Fig. 4A). Triple labeling of α-, β-, and PP-cells revealed a similar cytoarchitecture change (Fig. 4B).

### BrdU incorporation suggests cell proliferation is involved in α- and δ-cell regeneration

Rapid expansion of α-cells and δ-cells indicate that robust regeneration of non-β cells was induced after STZ-mediated β-cell destruction. Previous studies have demonstrated self-duplication, regeneration from precursor cells or trans-differentiation from other cells can all contribute to β-cell regeneration. Among these, both self-duplication and regeneration from precursor cells involve cell proliferation. To gain some insight into the mechanisms of non-β-cells regeneration, we first examined whether proliferation occurred during α- and δ-cell expansion using an *in vivo* BrdU incorporation assay. BrdU is a thymidine analogue that can be incorporated into the genome during DNA replication, and has been widely used as a marker for cell proliferation. In these experiments, the mice were provided with 1 mg/ml BrdU-containing drinking water immediately following STZ treatment. At different day post-STZ, the pancreases were harvested and subjected to immunohistochemical evaluation with an anti-BrdU antibody and islet cell markers.

**Figure 5:**
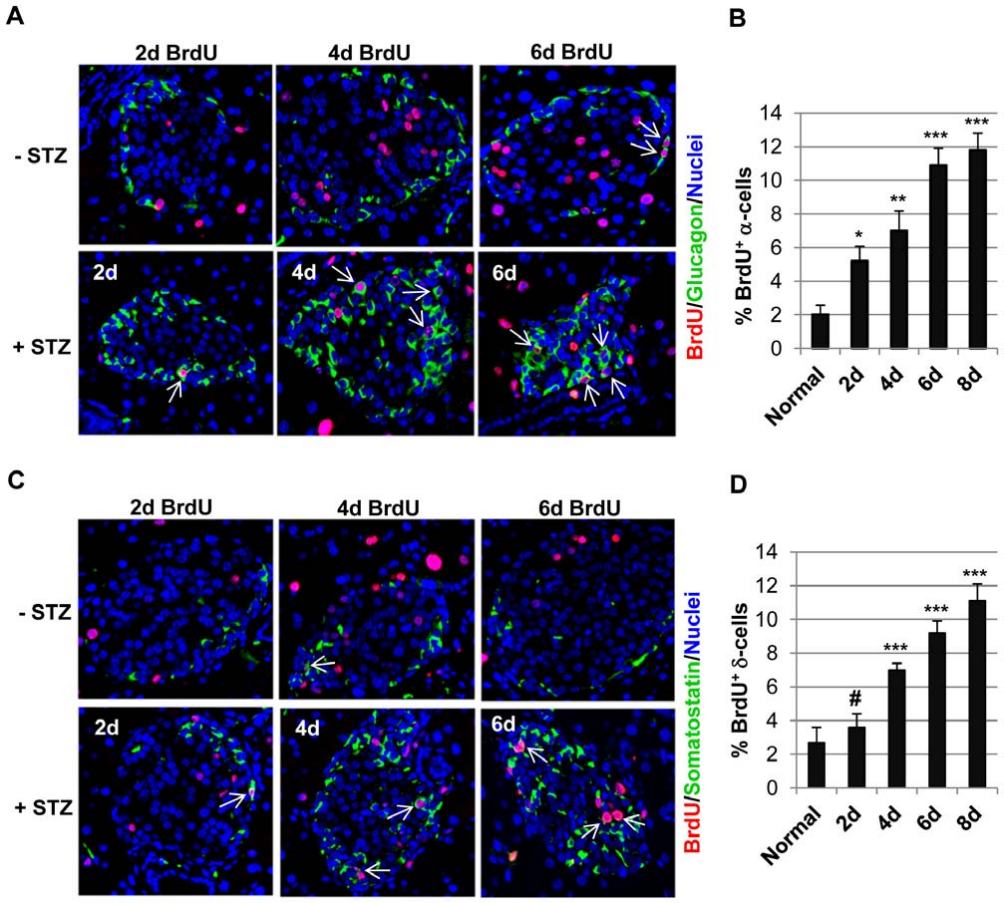
BrdU incorporation demonstrating that cell proliferation was involved in non-β-cell regeneration. Following STZ injection, 1 mg/ml of BrdU was added into the mice's drinking water. Normal mice were included as the control. At different day post-STZ, the pancreases were processed for immunofluorescence staining with anti-BrdU (red, A and C) and anti-glucagon (green, A) or anti-somatostatin (green, C). The arrows mark BrdU^+^ α-cells (A) and BrdU^+^ δ-cells (C) in the representative islets. The percentage of BrdU^+^ α-cells (B) and BrdU^+^ δ-cells (D) were calculated by dividing the number of these cells by total α- and δ-cells in each islet, respectively. Unpaired t-tests were performed to compare whether the percentage at each time point following STZ treatment was significantly different from that in normal mice. *: *p*<0.05, **: *p*<0.005, ***: *p*<0.0005, #: *p*>0.05 (not significant).

As shown in Fig. 5, in normal mice that were not treated by STZ, BrdU^+^ cells were detected in the core of the islets, which were composed of mainly β-cells. This is consistent with previous studies about β-cell regeneration [12], [21], [33]. Interestingly, we also detected BrdU^+^ α-cells and δ-cells in normal mice, although at very low frequency. This indicates, similar to β-cells, α-cells and δ-cells could also regenerate under normal physiological conditions (Fig. 5A and 5C). Following STZ treatment, α-cells and δ-cells expanded into the core of the islets. We could readily detect BrdU^+^ α-cells and BrdU^+^ δ-cells at each time point. Quantification of the percentage of BrdU^+^ α-cells (Fig. 5B) and δ-cells (Fig. 5D) showed STZ treatment significantly increased the proliferation of these non-β cells, supporting that proliferation was involved in the regeneration of these cells. It should be noted that a significant number of α-cells and δ-cells, including those that extended into the core area of the islets, were not labeled by BrdU. This could be explained by inefficient BrdU incorporation and/or failure of immunohistochemical staining, but did not rule out the possibility that these cells might have originated from non-proliferative mechanisms such as trans-differentiation of existing islet cells.

### Ki67 staining of proliferating cells argues for a role for self-duplication in the regeneration of α- and δ-cells

**Figure 6:**
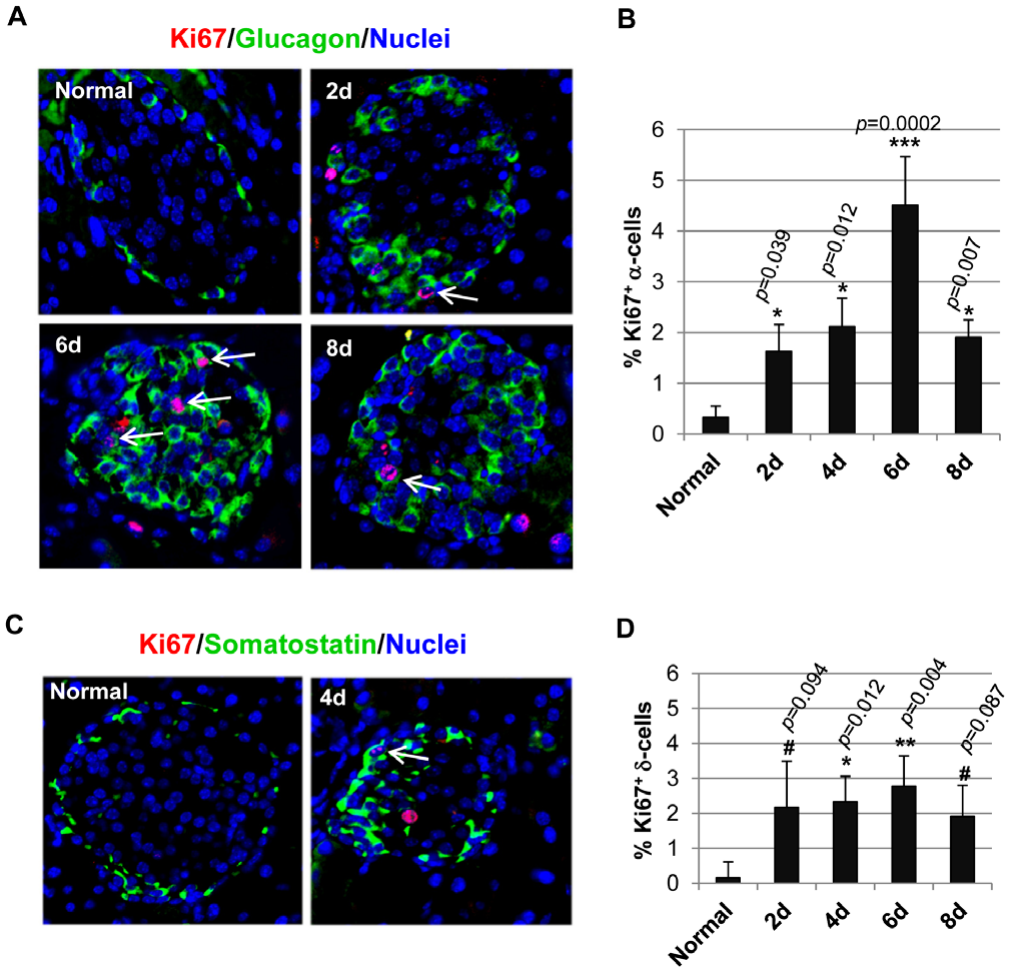
Ki67 staining highlighted proliferating α-cells and δ-cells. A and C: double staining of the islets with the proliferating cell marker Ki67 (red) with anti-glucagon (green, A) or with anti-somatostatin (green, C). The white arrows mark the proliferating α-cells and δ-cells, respectively. B and D: the percentage of Ki67^+^ α-cells/total α-cells (B) and Ki67^+^ δ-cells/total δ-cells (D) in each islet was quantified. Unpaired t-tests were performed to compare whether the data at each time point post-STZ was significantly different from that in normal mice. *: *p*<0.05, **: *p*<0.005, #: *p*>0.05 (not significant).

Since cell proliferation was involved in the non-β cell regeneration, next we examined whether self-duplication could be one of the proliferative mechanisms. To accomplish that, we stained the islet cells with Ki67, a marker for proliferating cells. Ki67 is expressed during the replicative phases of the cell cycle, and is thus an excellent marker for cells that are dividing. This is different from BrdU labeling because BrdU labels all cells that have undergone DNA replication and thus is an accumulative marker for cell proliferation. As shown in Fig. 6, at different days post-STZ treatment, we detected Ki67^+^ α-cells and δ-cells, which means these cells were in active replication. Quantitative analysis showed the percentage of replicating α-cells in STZ treated mice significantly increased compared to normal control, with the highest value achieved at Day 6 post-STZ (Fig. 6B). Similar observation was made for δ-cells as well, although the differences in the percentage of Ki67^+^ δ-cells were only significant at Day 4 and Day 6 post-STZ from normal control (Fig. 6D). Taken together, these data suggest α-cells and δ-cells could replicate, and self-duplication was one of the mechanisms for their regeneration following STZ treatment. Of note, similar to that found with the BrdU incorporation assay, the number of proliferating cells was far fewer than anticipated, especially compared to the robust regeneration observed. This may be attributable to poor antibody staining efficiency as the anti-Ki67 antibody was generated against human Ki67, and might not be efficient in detecting mouse Ki67. Nonetheless, the presence of proliferating α- and δ-cells argues for self-duplication as one of the mechanisms for their regeneration.

### Pdx1 staining indicates endocrine progenitor cells play a role in the regeneration of non-β cells

**Figure 7:**
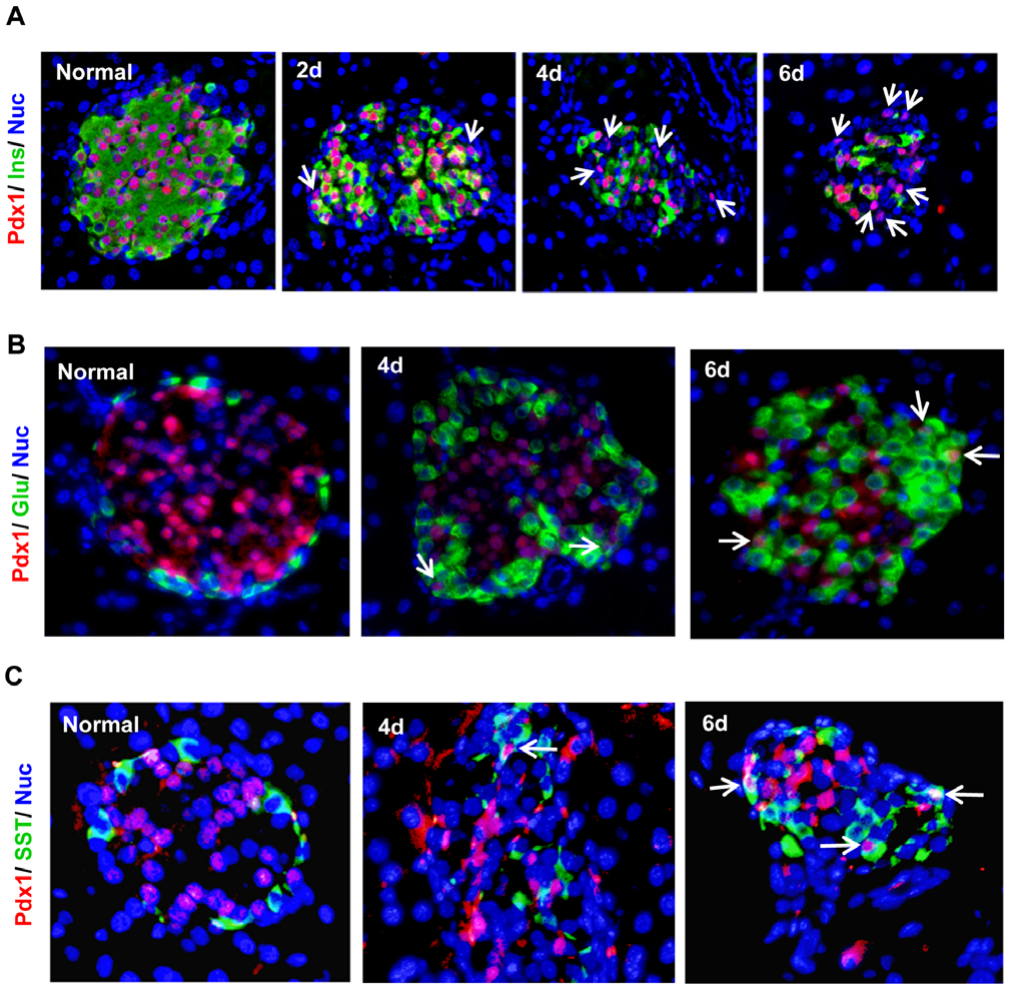
Pdx1 expression in STZ-treated mouse islets. The pancreatic islets from normal and STZ-treated mice were co-stained with anti-Pdx1 (red in A, B, and C) and islet cell markers including anti-insulin (A, green), anti-glucagon (B, green) and anti-somatostatin (C, green). In the normal islets, Pdx1 co-localized with insulin-expressing β-cells. Following STZ-mediated β-cell destruction, Pdx1 expression diminished, but became detectable in more and more non-β cells (A, arrows). Co-staining of Pdx1 with glucagon or somatostatin antibodies showed presence of Pdx1^+^/glucagon^+^ (B, arrows) and Pdx1^+^/somatostatin^+^ (C, arrows) cells following STZ-treatment, suggesting these cells are multipotent progenitor cells in transition to α- and δ-cells respectively.

Endocrine progenitor cells have been shown to play a role in the regeneration of β-cells [22], [23]. To examine whether they were involved in the regeneration of α- and δ-cells, we co-stained the pancreatic islet cells with Pdx1 antibody. The transcription factor Pdx1 plays a critical role in embryonic pancreas development, endocrine cell differentiation, and in maintaining mature β-cell function [34], [35], [36], [37], [38]. It is expressed in developing pancreatic precursor cells, and later becomes restricted to mature β-cells. Co-staining of Pdx1 and insulin showed Pdx1 was expressed in the β-cells of normal islets (Fig. 7A). In STZ-treated mice, Pdx1 expression diminished with β-cell loss, but subsequently, Pdx1 became readily detectable in more and more non-β cells (Fig. 7A). Since Pdx1 is a marker for β-cells or pancreatic progenitor cells, the Pdx1^+^/Ins^−^ cells represent the multipotent progenitor cells. The increasing presence of these cells suggests they regenerated following STZ treatment, and thus could play a role in the regeneration of the non-β cells.

In order to confirm these progenitor cells were involved in the regeneration of the non-β cells, we attempted to detect the cells that were in the transition process from progenitor cells to mature α-cell or δ-cells. Normally Pdx1 expression is not detected in mature α- or δ-cells. During transition from progenitor cells to α- or δ-cells, Pdx1 expression diminishes while glucagon or somatostatin expression turns on. Co-staining of the islets using Pdx1 and glucagon or somatostatin antibodies revealed the presence of Pdx1^+^/Glucagon^+^ and Pdx1^+^/somatostatin^+^ cells following STZ treatment (Fig. 7B and 7C), suggesting these cells were transitioning into mature α- or δ-cells. Taken together, these results suggest endocrine progenitor cells played a role in the regeneration of the non-β cells.

## Discussion

In this study, we characterized the dynamic change of endocrine cell composition of pancreatic islets in adult mice that were rendered diabetic with a single dose of STZ. Our data suggested there was a rapid and robust α- and δ-cell expansion following STZ-mediated β-cell destruction, supporting our hypothesis that adult non-β endocrine cells could be regenerated. We further sought to define the mechanisms, specifically the cellular sources, of their regeneration. Our results demonstrated both self-duplication and regeneration from progenitor cells played a role in this process.

Regeneration of α-cells in adult mice has been observed in mice whose β-cells were destroyed by toxins such as diphtheria toxin (DT) and multiple low-doses of STZ [24], [26]. Together with our discovery, these studies have confirmed that α-cell regeneration can be triggered by β-cell loss, the key characteristic of type 1 diabetes in animal models and in human patients. Indeed, previous studies have shown α-cell composition increases in human type 1 diabetic patients and in type 2 diabetic patients with reduced number of β-cells [28], [29], [30], [39], [40], supporting α-cell regeneration having occurred in these patients. How β-cell loss triggers non-β cell regeneration, however, is not clear. Interestingly, a recent study by Liu et al. has shown both insulin and glucagon promotes α-cell proliferation in α-cell culture [29]. Whether this is true *in vivo* remains to be investigated.

Other novel discoveries made in this study include the regeneration of δ-cells and PP-cells. Our study demonstrated that, similar to α- and β-cells, adult δ-cells could also regenerate rapidly under certain circumstances. In fact, our data on the mechanisms of α- and δ-cell regeneration indicate these specialized endocrine cells share similar mechanisms for regeneration. Although we did not quantify the expansion of PP-cells due to bi-hormonal staining of PPP and glucagon (Fig. 4), PP-cell expansion is clear. The nature of the bi-hormonal cells remains to be determined. One plausible explanation is they represent certain intermediate/precursor cells.

Over the past few years, significant progress has been made with regard to the regeneration of β-cells in adults. It has been demonstrated that β-cells can be regenerated by self-duplication, from progenitor cells or by trans-differentiation of α-cells [19], [20], [21], [22], [23], [24]. Mechanisms of non-β cell regeneration, however, have not been reported. In our initial search of the mechanisms for α-cell and δ-cell regeneration, we first examined cell proliferation, which underlines two of the mechanisms of β-cell regeneration: self-duplication and regeneration from progenitor cells. Our BrdU incorporation assays confirmed cell proliferation played a role in α- and δ-cell regeneration following STZ treatment. Nonetheless, the number of BrdU^+^ cells fell short compared to the massive increase of α- and δ-cells. Inefficient incorporation or staining defects may have accounted for the observed results. To exclude the possibility in regards to imminohistochemical staining, we performed the assays under different conditions and with different antibodies, and repeated the experiments many times. All showed similar results. In addition, the positive staining was very clean and clear (Fig. 5), suggesting that staining was not the issue. The BrdU concentration used in this study was similar to what has been reported in the literature [21], [33], [41], thus it was expected to be sufficient to label most, if not all, proliferating cells. It is possible, however, that the proliferation happened so rapidly that the concentration of BrdU was not sufficient for all of the proliferating cells to incorporate it into their replicating DNA. Another plausible explanation is this inability to label most newly formed α- and δ-cells simply mean mechanisms that do not involve proliferation played a part in their regeneration.

Although the number of BrdU^+^ cells was less than expected, our data demonstrated the participation of cell proliferation in the regeneration of α- and δ-cells. Furthermore, Ki67 labeling of proliferating cells showed both α-cells and δ-cells had the capacity of self-duplication, similar to β-cells [19], [20], [21]. Self-duplication is believed to be the major mechanism for normal β-cell turnover under physiological conditions. Our data suggest α- and δ-cells in adults may adopt the same mechanism for normal tissue replenishment. Self-duplication occurred in the diabetes models induced by a single-dose of STZ. However, the sparseness of Ki67^+^ α-cells or δ-cells, in contrast to their rapid and robust regeneration, indicates self-duplication may not be the major mechanism of regeneration following STZ-mediated acute β-cell destruction.

The transcription factor Pdx1 is expressed in developing endocrine cells during mouse embryogenesis and in mature insulin-producing β-cells [42]. β-cell specific inactivation of the transcription factor Pdx1 resulted in β-cell loss and α-cell expansion [34], [35]. Our examination of Pdx1 expression showed a diminishing of Pdx1 in β-cells, and an emergence of Pdx1^+^ non-β cells (Pdx1^+^/Ins^−^) following STZ treatment. These Pdx1^+^/Ins^−^ cells appeared to be multipotent endocrine progenitor cells which have the capacity to differentiate into α-, δ- and β-cells, mimicking embryonic endocrine cell development. Indeed, we were able to detect Pdx1^+^/glucagon^+^ and Pdx1^+^/somatostatin^+^ cells following STZ treatment, suggesting these cells were in the transition process into mature α- and δ-cells, respectively. Due to the transient nature of the transition, it is not unexpected that most of the glucagon^+^ and somatostatin^+^ cells were not Pdx1^+^. Taken together, our data demonstrate that endocrine progenitor cells play a role in the regeneration of α- and δ-cells. This is consistent with previous studies that have shown progenitor cells are present in adult mouse islets, and can serve as the cellular origins for β-cell regeneration [22], [23].

Another interesting discovery is, α-cell regeneration in our single dose model (130 mg STZ per kilogram bodyweight) occurred at a much more rapid pace compared to that in the multiple low dose STZ model (40 mg STZ per kilogram bodyweight daily for 5 days) [26]. In the single high dose model, significant α-cell regeneration was detected at Day 2 and peaked at Day 6 post-STZ. In contrast, an increase in α-cell composition was detected at Day 14, and did not reach the highest value until Day 21 in the multiple low-dose model [26]. This pace appears to correlate with the rate of β-cell loss and diabetes development in the two different STZ models— hyperglycemia develops by Day 2 for the single high dose model (Fig. 1), but it took 7 days in the multiple-low dose model [26].

Despite the rapid increase of α-cells in the STZ-treated mice, serum glucagon levels did not completely correlate with the number of α-cells. Glucagon levels decreased at first, and then gradually rose. This was not unexpected as hormonal secretion in the endocrine cells is known to be highly regulated. Normally, glucagon is secreted in response to low blood glucose, and is also regulated by serum insulin and somatostatin [13], [14], [15], [17], [18]. The serum glucagon levels were lower in STZ-treated mice, probably because of a higher blood-glucose than in normal mice. However, with the disturbances in insulin secretion and loss of normal cell-cell communication that resulted from β-cell loss [13], and α- and δ-cell expansion, glucagon secretion was no longer tightly regulated. Therefore, with an increasing presence of α-cells from Day 2 to Day 8, glucagon secretion increased despite the higher blood glucose in these STZ-treated mice.

It is noteworthy that α-cell hyperplasia has also been observed in neonatal rats following STZ treatment [43]. Similar to our study, α-cell and δ-cell composition increased during the first few days in the neonatal rats following a single diabetes-inducing dose of STZ. However, there are clear differences between neonatal and adult animals. For example, the neonatal rats developed hyperglycemia two days post- STZ treatment, but returned to near-normal level after 20 days [43], [44]. In contrast, the adult mice in our study could only survive two weeks without insulin treatment. In the neonatal rats, the islet architecture appeared to be normal with a dense β-cell core at Day 20 [43], while the islet cells in adults became intermingled following STZ treatment (Fig. 4). The differences are not surprising because neonatal pancreas undergoes substantial remodeling [7], [8], [9]. At this stage, β-cell apoptosis, replication, and neogenesis happen rapidly, and thus STZ-induced β-cell loss can be compensated.

In summary, our study has revealed that glucagon-producing α-cells and somatostatin-producing δ-cells can rapidly regenerate following a single diabetes-inducing dose of STZ treatment in adult mice. Both self-duplication and regeneration from intra-islet progenitor cells play a role in their regeneration. Our data, however, did not exclude non-proliferative mechanisms in the regeneration of the non-β cells.
